# ‘It’s a healthy choice’: a qualitative study of Australian consumers’ awareness and interpretation of nutrition-related claims on alcohol products

**DOI:** 10.1017/S136898002610278X

**Published:** 2026-05-25

**Authors:** Asad Yusoff, Alexandra Jones, Bella Sträuli, Paula O’Brien, Jacqueline Bowden, Simone Pettigrew

**Affiliations:** 1 Faculty of Medicine & Health, University of New South Wales, Australia; 2 Food Policy, The George Institute for Global Health, Australia; 3 School of Medicine and Dentistry, Public Health and Economics Modelling Team, Griffith University, Gold Coast, Australia; 4 The University of Melbourne, Melbourne Law School, Australia; 5 National Centre for Education and Training on Addiction, Flinders Health and Medical Research Institute, Flinders University, Australia

**Keywords:** Claims, Labelling, Qualitative, Alcohol

## Abstract

**Objectives:**

Alcohol companies are increasingly marketing products with nutrition-related claims to appeal to consumers. There are concerns around the potential of such claims to mislead; however, there is limited research on consumers’ attitudes to claims, why they use them and how they influence product selection. The aim of this study was to explore these issues among Australian consumers.

**Design:**

Nine online focus groups were conducted with approximately nine individuals per group (*n* 83). Groups were stratified by age, sex and drinking status. Participants were asked about their alcohol purchasing behaviours and presented with a selection of alcohol products that displayed nutrition-related claims (e.g. zero sugar).

**Setting:**

Australia.

**Participants:**

Australian adults who consumed alcohol at least twice per month.

**Results:**

Three major themes emerged from the data. First, most participants appeared to trust on-pack claims, viewing them as tools for making healthier choices. Claims were cited as being particularly useful when placed on the front of packaging to assist the rapid identification of products perceived as ‘healthier’ options. Second, participants appeared to use claims primarily to identify nutrients or ingredients they wanted to avoid, especially sugar, calories and preservatives. Finally, most participants supported the use of claims, with few raising concerns about them being potentially misleading.

**Conclusion:**

The results of this study add to growing evidence indicating that nutrition-related claims can mislead consumers about the healthiness of alcoholic beverages. Policymakers should seek to restrict the use of claims while also working to increase awareness of the health risks associated with alcohol consumption.

To appeal to health-conscious consumers, alcohol companies are developing products that are marketed with a variety of ‘better for you’ claims^(1)^. These claims can be defined as any on-pack text (excluding the Nutrition Information Panel and ingredients list) that provides information about any actual or purported health-related characteristics of the product^(2)^. Claims can include references to a wide range of factors such as nutrient content (e.g. low sugar), energy content (e.g. 90 cals), processing practices (e.g. organic) and ingredients (e.g. contains real fruit juice). The emergence of ‘better for you’ marketing has been interpreted by researchers, public health groups and industry analysts as a response to falling rates of alcohol consumption, particularly among younger drinkers^(3–5)^. This decline has been driven by several factors including a shift towards healthier lifestyles among the general population, a rise in the sober curious movement and an increasing awareness of the risks associated with alcohol consumption^(3,6)^.

On-pack claims can pose a concern for public health as they may divert consumers’ attention from the harms associated with alcohol consumption via the health halo effect^(1)^. This phenomenon occurs when consumers overestimate the healthiness of a product due to the presence of health-related information on product packaging (e.g. nutrient content claims)^(7)^. In the context of alcoholic beverages, the presence of claims may lead consumers to incorrectly believe that products featuring claims are healthier or less harmful than those without claims^(8,9)^. This misperception may arise due to claims diverting one’s attention away from the alcohol content of a product and towards other features. This is concerning as ethanol is carcinogenic and the most energy dense component of an alcoholic beverage^(10)^.

Globally, the regulation of ‘better for you’ claims on alcohol labels is limited. At present, the Codex Alimentarius Commission, the international food standards agency that issues guidance for nutrition labelling, does not yet provide alcohol-specific labelling guidelines^(11)^, leaving countries without a clear global standard to draw upon when developing national regulation. In the European Union and the United Kingdom, nutrient content claims are banned on the basis that the health risks associated with alcohol mean it should not be marketed on its nutritional qualities^(12,13)^. Canada and the United States permit the use of all types of nutrient content claims (provided there is an accompanying statement of average analysis or a Serving Facts statement)^(14,15)^, while in Australia such claims are allowed but only for sugar, carbohydrate, energy and gluten content^(16)^. Globally, other types of ‘better for you’ claims, such as those emphasising the absence of certain ingredients (e.g. No additives), natural qualities (e.g. Natural flavours) and production methods (e.g. Organic), are not explicitly regulated and are therefore permitted to be used on alcohol products.

Despite evidence that a significant proportion of alcohol products in some countries (e.g. Australia and Canada) feature claims^(17–19)^, research on consumer responses and attitudes towards ‘better for you’ claims remains limited. Existing studies have primarily used experimental designs to examine how claims influence product perceptions, showing that claims related to sugar, carbohydrate and energy content can increase the perceived healthiness of alcoholic beverages and reduce the perceived risk associated with consumption^(8,20,21)^. The few available qualitative studies have typically focused on younger age groups and found that ‘low-carb’ claims on alcoholic beverages may enable consumers to rationalise unhealthy choices^(22,23)^, and that young women are often drawn to products with low-sugar and low-energy claims because they align with ideals relating to female body image, such as the desire to be thin^(9)^. Research on other types of ‘better for you’ claims commonly displayed on alcohol products is scarce; however, evidence from other unhealthy products such as cigarettes and soft drinks has shown claims like ‘natural’ and ‘organic’ can mislead consumers into believing the products are healthier than products without claims, thereby influencing their choices^(24,25)^.

Examining how the different types of claims used by alcohol companies may shape consumer behaviour is important due to the apparent growth of ‘better for you’ marketing^(5)^ and the potential for such messaging to mislead consumers about the healthiness of alcohol^(8,21)^. Given the available quantitative evidence of the effects of nutrition claims on consumers’ perceptions of alcohol products^(8,20,21,26)^, further qualitative evidence is needed to better understand the mechanisms through which claims can influence perceptions of product healthiness. This information can assist policymakers to design effective strategies to counter potentially misleading on-pack messaging.

The context of the present study was Australia, where annual per capita consumption is high at around 10 litres of pure alcohol per person aged over 15 years^(27)^. Recently, the alcohol labelling regulator, Food Standards Australia and New Zealand, formally permitted the use of sugar claims (e.g. Sugar-free) on alcoholic beverages^(28)^. This decision was partly in response to the common use of these claims in the marketplace and industry arguments that it was too late to enforce a ban on them^(28)^. This has heightened existing concerns about the potential for these (and other) claims to signal that there is a ‘healthier’ way to consume alcohol^(29,30)^. Accordingly, the aims of this paper were to explore (i) Australian consumers’ attitudes to a variety of on-pack claims commonly featured on alcohol products and (ii) why and how they use these claims to inform their consumption decisions.

## Methods

The focus groups were part of a larger project exploring consumer attitudes towards alcohol and alcohol-related policies. Ethics approval for the study was granted by a University Human Research Ethics Committee.

### Participants

Eighty-three participants were recruited in March 2024 from three Australian states (New South Wales, Western Australia and Victoria) by two social research agencies (Thinkfield and ChitChat). Eligibility criteria required participants to be aged ≥ 18 years and consume alcohol at least twice per month. The sample size was determined a priori, informed by previous empirical evidence on achieving saturation in qualitative studies^(31,32)^. Before attending, participants were asked to report their usual alcohol consumption to determine whether they exceeded the Australian drinking guidelines of no more than 10 standard drinks per week and no more than 4 standard drinks on any single day^(33)^. As shown in Table 1, quotas were applied to ensure equal representation of participants by age groups and sex. However, no quotas were set for drinking status, resulting in an over-representation of participants who exceeded the drinking guidelines (approximately two-thirds of the sample compared with one-third of the Australian adult population^(34)^). Prior to participation, all individuals provided written informed consent, and upon completion of the focus group received reimbursement of $90.

**Table 1.** Sample profile (*n* 83) Table 1 long description.

	*n*	%
Gender		
Women	42	51
Men	41	49
Age		
18–30 years	28	34
31–50 years	26	31
51+ years	29	35
Drinking status^*^		
Complied with the Australian drinking guideline	27	33
Exceeded the Australian drinking guideline	56	68
Total	83	100

* Guideline: no more than four drinks on any single occasion and/or no more than ten drinks per week^(34)^.

### Data collection

Nine online focus groups were conducted with an average of nine participants in each session (range 8–10, *n* 83). To examine differences in responses by age, sex and drinking status, the groups were segmented using a matrix design (see online Supplementary materials Table S1 for the composition of each group). In line with best-practice recommendations for online focus groups^(35)^, the sessions lasted approximately 90 min, which was sufficient to explore the topics of relevance to the study.

Each group followed a semi-structured approach including unprompted and prompted discussion sections (the interview topic guide is provided in the online Supplementary materials). In the initial unprompted section, participants were asked about their alcohol purchasing patterns, including their preferred beverage type, usual purchasing locations and factors that influence their decision-making. This enabled mention of ‘better for you’ claims to emerge spontaneously. Participants were then shown images of five real-world products that featured a variety of ‘better for you’ claims (images shown in the online Supplementary materials). The products represented major brands sold in the Australian market and included a variety of product categories to reflect preferences across age and gender groups (wine, beer and premix products)^(36)^. Claims present on the depicted products related to sugar, carbohydrate, calorie, additive, gluten and fruit content; inclusion of natural flavours and vegan suitability.

With participants’ consent, the sessions were video recorded using Microsoft Teams. The audio recordings were subsequently split from the video and sent to an ISO-certified agency for transcription.

### Data analysis

NVivo qualitative data management software was used to code and analyse the data. Due to the limited prior evidence on attitudes towards ‘better for you’ claims on alcohol products, an emergent coding framework was developed. As per previous approaches to exploratory research, a single coder analysed the entire dataset using the constant comparative method^(37)^. First, the transcripts were analysed via an open coding approach to uncover patterns in the raw data and identify broad similarities and differences in participants’ attitudes and responses to various ‘better for you’ claims. These patterns were organised as codes that captured the most salient ideas in the raw data. Second, axial coding was undertaken to develop connections between the identified codes and create meaningful categories describing participants’ responses. Finally, all axial codes were analysed and grouped into core themes and sub-themes via selective coding. The single coder was a 27-year-old male with 3 years’ experience in alcohol research. To minimise bias, three of the authors were present at all the focus groups, enabling detailed discussions of the emerging themes in the context of the observed conversations. Prior to finalisation, all themes were reviewed and approved by the broader research team.

## Results

Three themes were identified from the data: (i) perceived credibility of claims, (ii) motivations for using claims and (iii) general support for the provision of claims. Each theme is discussed below, accompanied by relevant excerpts from the focus groups. For a summary of the themes and sub-themes please refer to Table 2.

**Table 2.** Summary of themes with illustrative quotes Table 2 long description.

Theme	Sub-theme	Example quotes
Claims perceived to be credible	Useful source of information	I do try and make sure I read the labels and I do get drawn to the low carb *(Woman, 51+).* Just with other health related information, I know sometimes in my life I will look at lower calorie beers or I will see, if it’s a premixed drink, if there’s like a lot of sugar *(Woman, 18–30).*
	Front of pack placement provided convenience during product selection	Yeah, it’s pretty eye catching. It’s good if that’s what they’re going for, it’s definitely noticeable and it will catch people’s eyes. I think it’s a good thing to have *(claims on the front)* and would definitely make me consider purchasing it, that’s for sure *(Man, 18–30).* …it’s actually putting it upfront, so you see it in the fridge display, similar if you’re reading everything upfront as opposed to having to pick it up, turn it around, twist it out and boom *(Man, 31–50).*
	Scepticism by some participants	I have a thing about artificial sweeteners, so whilst it says zero sugar I’m not a real sugary drinker, I would question what is making some sort of sweetness to it *(Woman, 51+).*
Reasons for using claims	Identify ‘negative ingredients’ (e.g. sugar and preservatives)	*Participant*: I think it’s well and good for them to tell us the energy or the calories that’s in it but there’s a lot of other factors that I think are more important than calories when it comes to the contents of things that we’re drinking. *Facilitator*: What would you prioritise? *Participant*: I’d be looking at the sugar, at the sodium *(Woman, 51+).* Yeah, because I’m not too concerned about the alcohol content. It’s pretty much when I can stop drinking, but the energy thing is one thing that’s scary, because it could affect your heart rate *(Man, 31–50).*
	Consume alcohol in a healthier manner	They sell it *(alcoholic kombucha)* to a few health food shops and to venues and parties. They’re always apparently quite young people that want to have a healthy drink *v*. normal beer and wines at their functions *(Woman, 51+).* My daughter drinks it (alcoholic kombucha). The reason I think she takes it is, kombucha is easy on the intestine. She’s afraid that if she takes beer and stuff like that, she’ll have bloating, those kinds of things, the excess carbs, beer belly and stuff like that *(Woman, 51+).* Yeah, I was just going to say I’ve noticed it a lot more lately. There’s a hype around the low carb beers and seltzers are the new drinks that are in because they have a lower sugar count. You feel more hydrated when you drink them *(Woman, 31–50).*
	Consume ‘natural’ products	I would say preservatives, always. If I’m looking at the labels, then I’ll also look at preservatives, as well *(Man, 31–50).* I love the peach passion fruit, but I also want to know because it doesn’t say it’s all naturally sweetened. So I want to know if it’s the actual fruit *(Woman, 31–50).*
General support for the provision of claims	Align with current health trends	It’s what people are after today, low carbs, low sugar, low you know. Very informative *(Woman, 51+ years)*. Zero sugar is just standing out for appealing to those people or to the group of friends who are very mindful of the intake of sugar in there as well *(Woman, 31–50 years)*.
	Positive reaction to novel claims (e.g. Probiotics)	I could definitely see myself drinking this drink…I think kombucha’s really nice and if I can get drunk while drinking it, why wouldn’t I do it? *(Man, 18–30).* I think both drinks are being marketed as healthy drinks, the Kombucha, you know, gluten free, vegan, probiotic *(Woman, 51+).*
	Few concerned about potential to mislead	So yeah, I think it’s an illusion thinking drinking a beer with less carbs is healthy *(Man, 18–30).*

### Theme 1: Claims perceived to be credible

When asked about factors influencing their purchasing decisions, many participants spontaneously said they used claims. Most expressed the belief that claims on alcohol products are a credible source of information that can aid consumers during product selection. The most frequently mentioned claims related to sugar, carbohydrate, energy and preservative content. While sugar, carbohydrate and energy claims were cited by participants across all age groups and sexes, preservative claims were predominantly discussed by women in the older two age groups (31–50 and 51+ years). No differences in responses to claims were observed by drinking status.

*I tend to go for wines that are low in carbs and low in sugar (Woman, 51+ years).*

*I think how many carbs they’ve got is always pretty eye-catching. Probably something that’s low carb, a sign that you look at, or low calories is probably the other main one that catches my eye (Man, 18–30 years).*

*The other thing that quite often is there is the additives. Like the chemical additives are on the back of the label too, and I do tend to watch for those (Woman, 51+ years).*

For participants who reported trusting and using claims, the prominent placement of claims on the front of packaging appeared to act as a convenient shortcut that assisted swift purchasing decisions, bypassing the need for a detailed examination of the product. For the majority of these participants, claims appeared to be a substitute for more comprehensive information, such as is routinely provided on food products in the form of nutrition facts panels or ingredient lists, but is much less frequently available on alcohol products. This sentiment was particularly evident in the prompted section of the discussion. Additionally, despite claims being presented in inconsistent formats across the stimuli (e.g. ‘low carb’, ‘< 2 g carb’, ‘80 % less carbs’), they were generally perceived as easy to understand. Participants did not express confusion or question the meaning of claims presented using imprecise terms such as ‘low carb’ or ‘low gluten’ rather than specific numerical values (e.g. < 2 g carbs).

*I think everything you need to know is clear and concise. You know, that it’s 80 % less carbs straight away looking at it (Woman, 51+ years).*

*I think I get enough information off the front. I wouldn’t need to look at the back … The key thing is about the less carbs (Man, 31–50 years).*

*I like to buy apple cider, but it needs to say low carb. It’s just a lot easier if it says on the label at the front rather than having to read it at the back of the bottle (Woman, 51+).*

A few participants expressed scepticism about the quality of information provided in nutrition claims on the example alcohol product. These individuals were mainly concerned about sugar claims, commenting that products labelled ‘low sugar’ or ‘zero sugar’ may contain artificial additives to compensate for the reduced sugar content. These additives were perceived as being potentially harmful to health due to their unnatural and chemical nature. The absence of a list of ingredients on some of the depicted products seemed to amplify these concerns due to the uncertainty surrounding the types of sweetening additives used. In nearly all instances, scepticism about claims was expressed by women, with representation across all age groups.

*As soon as I see zero sugar, I almost kind of turn away because I’m like, what other chemicals are in there? (Woman, 18–30 years).*

*If there’s no sugar in it, then what’s actually making it sweet? Is it full of nasty chemicals that might be dangerous? We don’t know (Woman, 51+ years).*

*It does say zero sugar, so it is made with artificial sweetener… so I would avoid that. (Woman, 51+).*

### Theme 2: Reasons for using claims

Participants appeared to primarily use on-pack claims to identify ingredients or nutrients they wanted to avoid. Claims were exclusively considered in relation to health rather than other factors such as ethical or environmental issues. Three main stated reasons for avoiding certain ingredients or nutrients were identified. First, many participants spontaneously reported using claims to avoid ingredients they believed caused adverse side effects after consumption, with the most commonly mentioned being sugar and preservatives. High sugar content was identified as a cause of feeling sick after drinking, while preservatives were associated with headaches or a general sense of discomfort. When presented with example products displaying claims such as ‘low sugar’, ‘preservative free’ and ‘organic’, other participants also reported actively looking for such claims when purchasing alcohol. The majority of participants raising these concerns were women, with representation across all age groups. Notably, very few participants mentioned selecting products with lower alcohol content as a solution to avoiding negative side effects. These individuals appeared to choose lower-alcohol products to either facilitate socialising or avoid drink driving.

*I do look for the ones that don’t have as much (sugar) because I feel sick in my stomach when I have too much (Woman, 31–50 years).*

*If you’re having a lot of premixed drinks, and you’re drinking them with the full sugar content, then that tends to affect your health the next day (Man, 18–30 years).*

*Sometimes if I’m getting a white (wine), I try and get an organic white. I find that whites give me a really bad headache (Woman, 31–50 years).*

*I find it important (alcohol content) when you are drinking and planning on driving, so you’re only having one or two. It’s important to know how much you are consuming. (Man, 18–30 years).*

Second, for many participants – particularly those aged 51 years and above – products featuring nutrient content and energy claims appeared to be perceived as providing a means of consuming alcohol in a ‘healthy’ or ‘healthier’ manner. While these individuals recognised that alcoholic drinks are inherently unhealthy, they primarily appeared to associate this unhealthiness with sugar, carbohydrates and calories. By avoiding these elements, they felt they could maintain their alcohol consumption without the restrictions they believed would be necessary when consuming a standard product. Of the nutrients discussed, carbohydrates were mentioned most frequently, with several participants citing familiarity with and preference for the low-carb varieties of popular brands. Like participants concerned about short-term side effects, alcohol content was rarely raised as a means of consuming alcohol in a healthier manner.

*If you still like to have a full-strength beer, you can have this with way less carbs, less calories, and it still tastes like a beer. It’s low in carbs, low in sugar. As someone said, it’s practically good for you (Woman, 51+ years).*

*I think zero sugar stands out. I mean, Cruiser is notoriously known for being super high in sugar … If someone is looking to reduce their sugar intake, it’s definitely good for that. (Man, 18–30 years).*

*I would take notice of that because I don’t like to have high carbs, high sugar. I always look out for that sort of thing … It’s a healthy choice (Woman, 51+ years).*

*I was diagnosed late last year with insulin resistance, but I don’t want to give up drinking beers altogether so I have looked at the low carb ones (Woman, 51+ years).*

Third, a few participants, predominately women aged 31–50 years, expressed a preference for more ‘natural’ products. These individuals reported actively seeking out alcoholic beverages labelled as being organically produced, free from artificial ingredients (e.g. additives) or made with what they considered natural alternatives to ‘bad’ ingredients (e.g. natural sweeteners or flavours). This sentiment was raised both spontaneously and when prompted, reflecting a strong motivation among these participants to monitor what they took into their bodies by choosing products containing ingredients they perceived as trustworthy.

*I look at no preservatives because it’s chemical stuff that you don’t need. It’s stuff that you shouldn’t be putting in your body. So I’ve always been conscious of that kind of stuff. No additives, more natural stuff (Woman, 31–50 years).*

*There are a lot of preservatives in alcohol and for me, especially with white wine, I feel it (Woman, 31–50).*

*I see there are no additives and preservatives. I like seeing that, personally. (Man, 31–50).*

### Theme 3: General support for the provision of claims

Most participants expressed support for the inclusion of claims on products to help inform product selection. Claims were seen as aligning with broader health-conscious trends, such as reducing sugar or carbohydrate intake, and appealing to individuals with specific dietary preferences, including gluten-free or vegan. Although claims related to nutrient content were more frequently mentioned as being useful, there was general belief that claims relating to processing practices and dietary requirements were in alignment with shifting social ideals around health and wellness.

*People are trying to do less sugar, less carbs and I think that’s (80 % less carbs) probably an attractive eye catch (Woman, 51+).*

*For me personally, it probably wouldn’t influence me but for some people, gluten free or vegan is important to them and that’s what they look for (Man, 18–30 years).*

*…that’s why the low carb and the ultra-low carbs have come in, alcohol’s always been associated with putting on weight and not being able to get rid of… so I think they’re trying to go more the lightweight beer without the alcohol being changed (Man, 51+ years).*

Once prompted by viewing the example product images, many participants noted additional claims they had not previously mentioned. These included references to fermentation and probiotic content. Such claims appeared to resonate particularly with those familiar with health-oriented non-alcoholic beverages like kombucha and were most frequently highlighted by women, some of whom described them as eye-catching because they signalled benefits for gut health.

*Probiotic is great too for your tummy and naturally fermented, so I really like that (Woman, 31–50).*

*It gets your attention straight away on a shelf if it was there. Naturally fermented, that’s always good for you, but I think that normally with kombucha anyway (Woman, 31–50 years).*

*Kombucha, I know that’s what people drink for their stomach to be healthy. It’s more looking at people that are health freaks and healthy people that want to have a beer (Man, 51+ years).*

Few participants expressed concerns that claims might be misleading or acknowledged the potential for nutrition-related claims to divert attention from the health risks associated with alcohol consumption. Among those who did raise such concerns, particular attention was given to the possibility that health-oriented claims – especially those frequently displayed on products from the non-alcoholic sector – could appeal to children and/or encourage misperceptions about the healthiness of the drinks. However, of note is that none spontaneously identified counterbalancing information (e.g. warning labels) as a necessary addition to alcohol labels when asked about what messaging they would like to see included on product labels.

*‘I’d worry more about this as being something that a kid is going to look at and go, ‘It’s Kombucha. It’s healthy. It’s good for me. I’m doing the right thing’ … and it’s 6 % alcohol’ (Woman, 31–50 years).*

*You’re basically convincing yourself that it’s healthy, even though you’re drinking alcohol (Man, 18–30 years).*

*The low sugar one or the low carbs one definitely to me is a trick in your brain to make you drink without that much guilt (Woman, 31–50 years).*

## Discussion

Consistent with previous research, this study found that on-pack ‘better-for-you’ claims have the potential to create a health halo effect that may lead consumers to mistakenly believe that products with claims are healthier options^(9,22,23)^. The apparent belief among many study participants that ingredients such as sugar and preservatives were the primary harmful components of alcoholic beverages demonstrates how claims can divert consumers’ attention away from the health harms of alcohol and towards other product features. Furthermore, the limited number of participants who cited reducing their alcohol intake as a way to minimise the negative side effects of drinking supports previous research indicating that consumers are often unaware of the serious harms associated with even low levels of alcohol consumption^(38)^.

Among participants who reported using on-pack claims, a key finding was their reliance on front-of-pack claims to assess and identify products they wanted to consume. The placement of ‘better for you’ claims on the front-of-packaging is problematic as it may lead consumers to underestimate the risks associated with alcohol consumption. This may particularly be the case when health-related information (e.g. ABV%) is placed in low-visibility locations (e.g. back of pack). Previous research in the food domain has shown that rather than assessing products in detail, consumers frequently rely on claims as heuristic cues to evaluate product attributes such as healthiness^(39)^. Given that the front of product labels is typically reserved for marketing material such as claims^(40)^, consumers are often exposed to labelling environments that prioritise marketing messages over information about the risks associated with using the product. This demonstrates that current labelling practices may mislead consumers and underscores the need for regulatory interventions that ensure information that supports consumers to make informed choices is presented prominently at the point of purchase.

A novel finding of the present study was the misleading effect of claims referring to the inclusion of functional ingredients (e.g. probiotics) or processing practices (e.g. fermentation) that are perceived to support wellbeing. This aligns with broader trends in food marketing focused on developing and promoting products containing ingredients or made with processing practices purported to support wellness and prevent disease^(41)^. Although the participants in the present study did not spontaneously mention these claims, potentially due to their historical low prevalence in the alcohol market^(17)^, the evident familiarity and positive attitudes towards them seemed to reflect participants’ prior exposure to similar language on non-alcoholic beverages. While most nations prohibit alcoholic beverages from making health claims^(12–16)^, the use of terms such as ‘probiotics’ may nevertheless suggest health benefits. In light of the growing presence of functional beverages in the alcohol market (e.g. alcoholic kombuchas)^(17,41)^, these results highlight the need for additional research into the potential misleading effects of claims relating to functional ingredients to inform the development of policies to limit the promotion of beverages that are indirectly marketed as having health benefits.

A notable finding of the current study was the greater receptiveness to claims among women. This is concerning, as women tend to be more health conscious than men^(42)^, potentially making them more vulnerable to marketing tactics that portray certain alcoholic beverages as healthier choices. Women in this study appeared especially responsive to claims relating to additives and novel ingredients such as probiotics, which are often associated with wellness^(43,44)^. This aligns with previous research indicating that alcohol companies have strategically targeted women, leveraging prevailing social norms and values that associate female identity with health and self-care^(9,45)^. Such marketing tactics risk undermining public health messaging by aligning alcohol products with wellness ideals, potentially minimising women’s perceptions of the risks associated with alcohol consumption. These findings raise important concerns for public health, particularly given the rapid increase in alcohol consumption among women compared to men^(46)^, and in light of ongoing efforts to reduce alcohol-related harms that specifically affect women, such as fetal alcohol spectrum disorder and breast cancer^(38)^.

### Policy implications

The findings of this research highlight two potential policy implications for the Australian market. First, given the health halo effect that has been found to be caused by ‘better for you’ claims on alcohol products in this and prior research^(8,9,20–23)^, regulators could consider more tightly regulating the use of such claims to avoid consumers from being misled about product healthiness. Such a step would be in line with a core principle of the Codex Alimentarius Food Labelling Guidelines, which states that any pre-packaged food (including alcohol as per the Codex definition of ‘food’) should not be ‘described or presented on any label or in any labelling in a manner that is false, misleading or deceptive’^(47)^. Although an outright ban on the use of ‘better for you’ claims would be the most effective approach for protecting consumers, the findings of this study suggest that at a minimum, claims should be banned from appearing on the front and top of packaging where they are most likely to serve as cues for assessing a product’s overall healthiness.

A second implication is the need for policy measures to better inform consumers about the health risks associated with alcohol consumption. Previous research has shown that many consumers remain unaware of the long-term harms caused by alcohol^(48)^, and the findings of the present study further suggest that even the well-known risks (e.g. liver damage) are rarely considered when selecting alcohol products. To address these gaps, one strategy could be the introduction of mandatory health warning labels on products that inform consumers about the long-term harms of alcohol (e.g. cancer). Such labels have been shown to improve knowledge of the risks associated with alcohol consumption^(49)^, counteract the misleading effect of several types of ‘better for you’ claims^(26)^ and reduce intended consumption^(49)^. Furthermore, the provision of on-pack warning labels would support consumers’ right to know about the potential risks of using a product, thereby encouraging more informed decision-making at the point of purchase.

### Limitations

The primary limitation of this study was the modest sample size, and as such, the findings of this research can only be viewed as indicative. Further work is required to understand if the results are generalisable to the broader drinking population, including in other countries where attitudes towards claims may differ. Second, the reliance on a pre-specified sample size meant that data collection was not guided by theoretical saturation. As a result, the full range of perspectives on the use of claims on alcohol products may not have been explored. Third, individuals who exceeded the Australian drinking guideline were oversampled. This could have resulted in biased responses as heavier drinkers may be more susceptible to on-pack advertising^(50)^. Fourth, the use of a single coder may have introduced bias, although efforts were made to minimise this outcome through the attendance of multiple researchers at all the focus groups, enabling informed discussions about the emerging themes, along with conferral with the broader authorship team. Finally, while the strength of this study was the variety of claims explored, not all alcohol types were used as stimuli. Future research could investigate consumer responses to claims on cider and spirits products to better understand whether responses vary by alcohol type.

## Conclusion

The findings of this study contribute to the growing body of evidence showing that the presence of ‘better for you’ claims on alcohol labels can create a health halo effect. Despite their misleading nature, this study found that participants appeared to trust claims, perceived them as useful for decision-making and often used them to avoid ingredients they considered undesirable. The observed consumer receptiveness to claims relating to functional ingredients (e.g. probiotics), particularly among women, is concerning given the growing presence of functional alcoholic beverages and the apparent lack of regulatory oversight on this issue. These insights highlight the need for further work to develop regulatory frameworks that restrict the use of ‘better for you’ claims and prevent companies from promoting certain types of alcohol as healthier options.

## Supporting information

10.1017/S136898002610278X.sm001 Yusoff et al. supplementary material Yusoff et al. supplementary material

## Supplementary material

For supplementary material accompanying this paper visit https://doi.org/10.1017/S136898002610278X

## Table 1. Long description

The table presents the profile of eighty-three participants recruited in March 2024 from three Australian states: New South Wales, Western Australia, and Victoria. The table is divided into three main categories: gender, age, and drinking status. Under gender, there are 42 women, representing 51 percent of the participants, and 41 men, representing 49 percent. The age category is broken down into three groups: 18-30 years with 28 participants (34 percent), 31-50 years with 26 participants (31 percent), and 51+ years with 29 participants (35 percent). The drinking status category shows that 27 participants (33 percent) complied with the Australian drinking guideline, while 56 participants (68 percent) exceeded it. The table highlights an over-representation of participants who exceeded the drinking guidelines compared to the general Australian adult population.

Navigate back to Table 1..

## Table 2. Long description

A table with three columns labeled Theme, Sub-theme, and Example quotes, and multiple rows detailing themes such as Claims perceived to be credible, Reasons for using claims, and General support for the provision of claims. Each theme is further divided into sub-themes with corresponding example quotes from participants of different age groups and genders. The table highlights various perspectives on the credibility and usefulness of claims on alcoholic beverages, reasons for using such claims, and general support for the provision of these claims. Notable trends include the emphasis on health-related information, convenience of front-of-pack placement, and the desire to consume alcohol in a healthier manner.

Navigate back to Table 2..
