# Supplementary material for: ‘It’s a healthy choice’: a qualitative study of Australian consumers’ awareness and interpretation of nutrition-related claims on alcohol products
Source: Public Health Nutr. 2026 May 25;29(1):e131. doi: 10.1017/S136898002610278X (PMC13434554; doi:10.1017/S136898002610278X)
Supplement: Supplementary file 1 [file S136898002610278Xsup001.docx]

**Supplementary materials**

**Focus group topic guide**

**Unprompted discussion topics**

1. Beverage preferences and consumption patterns
   - Types of alcoholic beverages typically consumed.
   - Frequency and context of consumption.
2. Alcohol sourcing and purchasing behaviours
   - Common methods and locations for purchasing alcohol.
   - Typical purchasing habits.
3. Determinants of purchasing decisions
   - Criteria for selecting between different alcohol categories (e.g., beer vs. wine vs. spirits).
   - Criteria for selecting between different brands.
4. Engagement with product labels
   - Frequency and extent of label examination during purchase.
   - Types of information noticed and recalled.
   - Information perceived as valuable/influential.
   - Suggestions for additional information that would be useful on labels.
5. Health-related on-pack information
   - Awareness and recall of health-related messaging on alcohol product packaging.
   - Perceived relevance and influence on purchasing or consumption decisions.

**Prompted discussion topics**

*(Following exposure to selected product images. See below for images shown to participants)*

6. Reactions to label designs
   - Immediate impressions and interpretations of the presented product labels.
7. Attitudes toward ‘better-for-you’ claims
   - Perceptions of ‘better for you’ claims on alcohol packaging.
   - Credibility and appeal of such claims
   - Concerns regarding use of claims
   - Implications for various population sub-groups

**Example stimuli**

  

**Supplementary table S**1: Focus group matrix design

| **Age Group** | **Gender** | **Drinking status*** |
| --- | --- | --- |
| 18-30 | Men | Exceeds guidelines |
| 18-30 | Men | Within guidelines |
| 18-30 | Women | Within guidelines |
| 31-50 | Men | Exceeds guidelines |
| 31-50 | Women | Within guidelines |
| 31-50 | Mixed | Exceeds guidelines |
| 51+ | Women | Within guidelines |
| 51+ | Mixed | Exceeds guidelines |
| 51+ | Mixed | Exceeds guidelines |

*Guideline: no more than 4 drinks on any single occasion and/or no more than 10 drinks per week (1)

**Reference**

1. National Health and Medical Research Council. The Australian Government - National Health and Medical Research Council. 2025. Australian guidelines to reduce health risks from drinking alcohol. Available from: https://www.nhmrc.gov.au/health-advice/alcohol
